# Effects of supplementing a *Bacillus*-based direct-fed microbial on feed intake, apparent total-tract nutrient digestibility, growth rates, and metabolic responses of *Bos indicus* beef heifers during the dry and early vegetative seasons

**DOI:** 10.1093/tas/txag046

**Published:** 2026-04-22

**Authors:** Isabella R T Souza, Bruno I Cappellozza, Pedro B Domingues Neto, Leandro S Lima, Rafaela S Leite, Luiz Henrique L Fonseca, Roberto X Bolsanello, Natália Sega, Arquimedes Lima Júnior, André L N Rigueiro, Pedro Henrique Terêncio, Oscar C M Queiroz, Reinaldo F Cooke, Philipe Moriel, José L M Vasconcelos

**Affiliations:** Faculdade de Medicina Veterinária e Zootecnia, Universidade Estadual Paulista, Botucatu, SP 18618-970, Brazil; Novonesis, Lyngby 2800, Denmark; Faculdade de Medicina Veterinária e Zootecnia, Universidade Estadual Paulista, Botucatu, SP 18618-970, Brazil; Faculdade de Medicina Veterinária e Zootecnia, Universidade Estadual Paulista, Botucatu, SP 18618-970, Brazil; Faculdade de Medicina Veterinária e Zootecnia, Universidade Estadual Paulista, Botucatu, SP 18618-970, Brazil; Faculdade de Medicina Veterinária e Zootecnia, Universidade Estadual Paulista, Botucatu, SP 18618-970, Brazil; Novonesis, Valinhos, SP 13278-327, Brazil; Phibro Animal Health Corporation, Guarulhos, SP 07112-070, Brazil; Phibro Animal Health Corporation, Guarulhos, SP 07112-070, Brazil; TecnoBeef Indústria e Comércio S.A, Altair, SP 15430-000, Brazil; TecnoBeef Indústria e Comércio S.A, Altair, SP 15430-000, Brazil; Novonesis, Lyngby 2800, Denmark; Department of Animal Science, Texas A&M University, College Station, TX 77843, United States; University of Florida, IFAS—Range Cattle Research and Education Center, Ona, FL 33865, United States; Faculdade de Medicina Veterinária e Zootecnia, Universidade Estadual Paulista, Botucatu, SP 18618-970, Brazil

**Keywords:** *Bacillus* spp, forages, metabolism, nutrient digestibility, performance

## Abstract

This experiment evaluated intake, nutrient digestibility, growth rates, and metabolic responses of *Bos indicus* beef heifers fed *Bacillus* spp. when offered a low-quality tropical hay [≤5% crude protein (**CP**)] and then transitioned to lush pastures during the early vegetative stage. Nellore heifers (*n* = 48; 20 ± 1 mo) were ranked by initial body weight (**BW**; 312 ± 26.4 kg) and randomly assigned to Tifton 85 hay-based diet and a protein supplement offered at 0.30% BW with or without (**CON**; *n* = 24) the inclusion of a *Bacillus*-based direct-fed microbial (**DFM**; **BAC**; *n* = 24). Animals were housed individually throughout the experiment, resulting in 48 drylot pens. The experimental period lasted 140 d, while individual full BW and blood samples were collected every 28 d. In the last 3 d of each 28-d period, fecal grab samples were collected from all heifers to assess the apparent total-tract digestibility. After the dry season, heifers were transitioned into lush pastures for 15 d and offered the same treatments (total experimental period = 156 d). Dry matter (**DM**) availability was measured at the beginning and end of the grazing period, whereas BW and blood samples were collected over the 15-d period. All data were analyzed with SAS (version 9.4). During the dry season, no treatment effects were observed (*P* ≥ 0.20) for feed intake or ADG. Dry matter and CP digestibility were greater for BAC (*P* ≤ 0.05). Heifers fed BAC had greater blood urea-N (**BUN**) and plasma insulin-like growth factor-I (**IGF-I**; *P* ≤ 0.02), while mean serum glucose tended (*P* = 0.09) to be greater. Treatment × day effects (*P* < 0.01) were observed on BW change during the 15-d grazing season. The BAC-fed heifers tended to lose (*P* ≤ 0.09) more BW from day 0 to 1, but gained (*P* < 0.01) more BW from days 0 to 15 of the period. During the 15-d grazing period, heifers fed BAC had a greater overall BW change and average daily gain (**ADG**) when compared with CON cohorts (*P* < 0.01). No differences were observed for DM availability at the beginning of the grazing period (*P* = 0.61), whereas paddocks assigned to BAC heifers had less DM available after 15 d (*P* = 0.02). In summary, feeding *Bacillus* spp. increased DM and CP digestibility, serum hormones and metabolites associated with energy and protein metabolism in beef heifers fed a low-quality tropical hay, without effects on growth rates. When the heifers were transitioned to lush pastures for 15 d, *Bacillus* spp. supplementation improved the growth rate of the heifers, positively impacting the entire growth of the herd.

## Teaser

This study reported the benefits of a *Bacillus*-based direct-fed microbial on nutrient digestibility and metabolism of beef heifers fed low-quality hay, while also improving grazing performance of the same herd during the early vegetative period.

## Introduction

Forages represent the major feedstuff required and used in livestock operations, including cow-calf and stocker (Watson et al. 2015). Specifically for cow-calf and stocker operations based on the tropics, seasonal variation that subsequently impacts the quantity and availability of nutrients in the forages (Vendramini et al. 2023) may determine the need of a strategic supplementation, such as minerals, protein, and energy, to optimize nutrient utilization, forage intake, and herd performance throughout the year (McDowell and Arthington 2005). Hence, alternatives that maximize nutrient digestion and performance in cattle fed low-quality tropical grasses are warranted and should be studied. Direct-fed microbials (**DFM**), more specifically those based on *Bacillus* spp., may be a feasible nutritional tool to improve nutrient digestion and utilization in grazing beef cattle (Cappellozza et al. 2025). In fact, dry matter (**DM**), neutral detergent fiber (**NDF**), and crude protein (**CP**) digestibility have increased following the dietary inclusion of *Bacillus* spp. either in vitro or in vivo (Pan et al. 2022; Cappellozza et al. 2023; Limede et al. 2025; Souza et al. 2025), supporting the enzymatic activity of these strains (Rhayat et al. 2019; Luise et al. 2022) and the potential benefits it may bring to ruminants. However, to our knowledge, no other study evaluated the long-term supplementation of *Bacillus* spp. to cattle grazing low-quality tropical pasture.

Moreover, as the seasons change, the cow-calf operation that relies on grazing may not have the adequate nutritional balance in the supplement to meet the nutrients that are missing and/or reduce the ones that may be in excess when the rainy season starts, such as the case of protein. Nonetheless, understanding the performance, metabolic, and health impacts of excessive nutrient intake in specific periods of cattle grazing is paramount to provide technical advice to beef producers. Recently, Souza et al. (2025) demonstrated reduced severity of diarrhea cases, blood urea-N (**BUN**), and alleviated performance losses in *Bos indicus* beef heifers grazing Tifton 85 grass during early vegetative stage (25% CP) and offered a high-protein supplement containing a hydroxychloride source of trace minerals. Diarrhea in this setting is likely caused by either the excessive urea content of the grass or an imbalance of electrolytes (Here et al. 2024). Therefore, alternatives that alleviate the performance and health losses caused by heifers grazing forages during early vegetative stage, while optimizing N utilization, are warranted and should be evaluated. *Bacillus*-based DFM affected plasma metabolome and fecal microbiome of beef heifers (Izquierdo et al. 2025; Sousa et al. 2025), improved the integrity of intestinal epithelial cells (Boll et al. 2024a) and N utilization (Dias et al. 2022; Cordeiro et al. 2024), benefited the health of pre-weaning dairy calves (Magalhães et al. 2024), the immune response of stressed beef cattle (Mackey et al. 2024, 2025), and the performance of both dairy (Magalhães et al. 2024; Copani et al. 2025) and beef cattle (Izquierdo et al. 2024; Mackey et al. 2024; Moriel et al. 2025). However, no other study evaluated the effects of a *Bacillus*-based DFM on cattle offered lush pastures. Based on this rationale, we hypothesized that feeding a *Bacillus*-based DFM would improve nutrient digestion in beef heifers offered a low-quality warm-season hay, while benefiting the performance and metabolic responses of heifers grazing tropical forages during the early vegetative stage. Hence, our objectives were to evaluate (i) feed intake, nutrient digestibility, growth rates, and metabolic responses of *Bos indicus* beef heifers offered a low-quality warm-season hay (≤5% CP) and (ii) growth rates and metabolic responses of *B. indicus* beef heifers grazing forages during the early vegetative stage, following the supplementation of *Bacillus* spp.

## Materials and methods

This experiment was conducted at a commercial cow–calf operation located in São Manuel (Sítio Santo Antônio, São Paulo, Brazil), from June to December 2024, split between 140 d of dry season and 16 d of grazing. All heifers were managed following accepted practices and experimental protocols, approved by the Animal Ethical Use Committee of São Paulo State University (protocol #001/2024).

### Dry season

*Animals and diets.* On day-14 of the study, Nellore (*n* = 48) heifers (age = 20 ± 1 mo) were randomly assigned to 1 of 48 individual drylot pens (10 × 25 m) and provided free choice access to Tifton 85 (*Cynodon dactylon*) hay and mineral salt from days -14 to -1. Throughout this period, individual hay dry matter intake (**DMI**) was measured daily.

Individual full body weight (**BW**) was recorded on days -1 and 0. Then, heifers were ranked according to the mean initial BW (312 ± 26.4 kg) and randomly assigned to 1 of 2 treatments (*n* = 24 heifers/treatment), which consisted of a Tifton 85 hay-based diet and a protein supplement (Tecnophós Proteico EN-25; TecnoBeef Indústria e Comércio S.A., Altair, SP, Brazil; DM basis) offered at 0.30% BW with or without (**CON**; *n* = 24) the inclusion of a *Bacillus*-based DFM (**BAC**; Bovacillus, Novonesis, Valinhos, SP, Brazil; *n* = 24). The BAC contained a proprietary mixture of *Bacillus licheniformis* 809 and *B. subtilis* 810, which was added at a dosage of 1.8 g/kg of the protein-based supplement (5.8 × 10^9^ colony forming units/kg of concentrate), following the recommendations of the manufacturer. The protein-based supplement contained citrus pulp, peanut meal, wheat meal, limestone, urea, sodium monensin, and a mineral-vitamin mixture. The nutritional profile of the Tifton 85 hay and protein-based supplement is presented in Table 1.

**Table 1:** Nutritional profile (mean ± SEM) of the tifton 85 (*Cynodon* sp.) hay and the protein-based supplement offered throughout the dry season of the study^a^.

Item	Tifton 85 hay	**Supplement** ^b^	
		CON	BAC
**Dry matter (DM), %**	91.1 ± 1.87	91.1 ± 0.64	92.1 ± 0.59
**Crude protein, % DM**	4.83 ± 0.386	25.3 ± 0.37	26.0 ± 1.97
**Neutral detergent fiber, % DM**	75.4 ± 2.10	26.0 ± 1.58	27.2 ± 1.91
**Acid detergent fiber, % DM**	46.3 ± 1.48	14.7 ± 1.62	14.3 ± 1.24
**Hemicellulose, % DM**	29.1 ± 0.81	11.3 ± 0.37	13.0 ± 1.29
**Lignin, % DM**	8.50 ± 1.864	6.69 ± 0.932	7.01 ± 1.60
**Cellulose, % DM**	37.8 ± 1.254	8.05 ± 1.329	7.24 ± 0.600
**Ash, % DM**	4.97 ± 0.422	24.1 ± 2.27	24.9 ± 0.59

a Hay and supplement samples from each treatment were collected every 28 d and analyzed separately throughout the experimental period for the determination of the nutritional profile.

b The mineral composition included 11.6 to 51.6 g/kg of Ca, 6,500.00 ppm of P, 65 ppm of F, 39 g Na, 3,000 ppm of Mg, 8,000 ppm of S, 170 ppm of Mn, 330 ppm of Zn, 100 ppm of Cu, 6 ppm of Co, 2 ppm of Se, 6 mg I, and 150 ppm of sodium monensin.

In the dry season, the experimental period lasted 140 d and was divided into 5 periods of 28 d each for sample collection and adjustment of the supplement offer. All CON and BAC heifers were fed the pre-determined amount of the protein-based supplements (0.30% of BW; DM basis) once daily (0730 h), followed by the hay feeding at approximately 0800 h. The protein supplement and hay were offered in separate feed bunks. All heifers were fed the hay in amounts to ensure ad libitum intake and any orts of the supplements and hay were recorded in the following morning, prior to the treatment and hay feeding.

*Sampling and laboratorial analyses.* On day 25 of each 28-d period, the particle size distribution of the hay offered to each heifer was measured from each pen with the Penn State Particle Separator (**PSPS**), according to the methodology previously described by Kononoff and Heinrichs (2003).

Full individual BW was recorded every 28 d throughout the 140-d experimental period to determine BW change and to adjust the amount of concentrate to be offered in the next 28-d period. The adjustment of the concentrate was performed by calculating the average daily gain (**ADG**) of the animals in the current period and concurrently estimating the average BW in the following 28-d period, so that the supplement offer would be as close as possible to 0.30% BW. Dry matter intake (**DMI**) of the supplement, hay, and supplement + hay was recorded daily throughout the experimental period, whereas samples of the offered and non-consumed hay were collected daily from each individual pen and dried in the microwave for dry matter (**DM**) calculation (Oetzel et al. 1993). In rainy days, sampling of the orts was not performed. Moreover, hay and supplement samples (CON and BAC) were collected on days, 26, 27, and 28 of each period and analyzed for their nutritional profile (Table 1).

In the morning (between 0700 and 0800 h) and afternoon (between 1600 to 1700 h) of days 26, 27, and 28 of each period, fecal grab samples were collected from all animals for determination of the apparent total-tract digestibility (**ATTD**), following the methodology described by Polizel et al. (2020). The fecal material was sampled and immediately stored at −20 °C for subsequent laboratory analysis. Frozen samples were then shipped to a commercial lab (Instituto Laboratorial de Alimentos e Nutrição Animal, Pompéu, MG, Brazil) for the laboratorial analysis. The fecal DM composition was determined by drying the samples in an oven at 105 °C for 24 h and ash content was determined by burning the samples in a muffle furnace at 550 °C for 4 h (AOAC 1997). Total nitrogen (**N**) determination was performed using a Leco FP-528 (Leco Corporation; Saint Joseph, MI), according to the methodology proposed by AOAC (1997), whereas NDF content was analyzed following the procedures described by Van Soest et al. (1991) with the addition of thermostable α-amylase and sodium sulfite in an Ankom-200 (Ankom Tech Corp., Fairport, NY). Following NDF determination, acid detergent fiber (**ADF**) was evaluated (Goering and Van Soest 1970) in an Ankom-200 (Ankom Tech. Corp.) and lignin was determined by the methodology described by Ellis et al. (1946). Then, hemicellulose was determined by subtracting NDF and ADF, whereas cellulose was determined by the difference between ADF and lignin (NASEM 2016). Apparent total-tract digestibility was calculated according to the formula: ATTD (%) = ((DMI × NCDM) – (FDM × NCFM) × 100)/(DMI × NCDM), where ATTD = apparent total-tract digestibility, DMI = dry matter intake, NCDM = nutrient content of the DMI (%), FDM = fecal dry matter, and NCFM = nutrient content of the fecal DM (%).

Concurrently with the determination of the individual BW on day 28 of each period, blood samples were collected into commercial blood collection tubes (Vacutainer, 10 mL; Becton Dickinson) containing no additive. Blood samples were placed immediately on ice after collection, centrifuged (2500 × *g* for 30 min; 4 °C) for serum harvest, and stored at −20 °C on the same day of sample collection. Serum samples were analyzed for haptoglobin (Cooke and Arthington 2013), insulin-like growth factor-I (**IGF-I**; #SG100; R&D Systems, Inc., Minneapolis, MN; Cooke et al. 2012), blood urea-N (**BUN**; BS200E Veterinary Chemistry Analyzer; Mindray, California, USA), and glucose (respons®910VET Veterinary Chemistry Analyzer; DiaSys Diagnostic Systems USA LLC, Wixom, MI; Colombo et al. 2021). The intra- and inter-assay CV for these analyses were ≤ 7.5%.

### Grazing (early vegetative) season

To reproduce and, potentially mimic, the physiological challenges of grazing warm-season forages at the early vegetative stage, Tifton 85 (*Cynodon* sp.) pastures were used (*n* = 24 paddocks/treatment; 1 heifer/paddock), similarly to what has been adopted as a management procedure by Souza et al. (2025). Following the management on day 140 of the study, all heifers were managed in the paddocks for a 15-d grazing period. Briefly, approximately 21 d prior to the beginning of the early-vegetative period, all paddocks were mowed to ground level and fertilized with 112 kg of N and 64 kg of K per hectare to stimulate forage regrowth. This approach was intended to generate sufficient herbage mass and allowance that exceeded the threshold that limits cattle forage intake (Inyang et al. 2010). On day 0, heifers, within each respective treatment (CON and BAC), were individually allocated to pastures for a grazing period of 15 d, while also receiving the same protein-based supplement (Tecnophós Proteico EN-25; TecnoBeef Indústria e Comércio S.A.) daily at 0800 h and at the same amount (0.30% BW). This approach was used to mimic what often happens in commercial settings when the rainy and, consequently, early vegetative stage starts in the tropics and the supplementation program may not be properly adjusted and in accordance to the novel environmental/seasonal situation.

*Sampling and laboratorial analyses.* At the beginning and end of the 15-d grazing period, the herbage mass was determined by hand-clipping forage to ground level inside a 1 m^2^ quadrant (20 random locations within each pasture), as described by Fieser et al. (2007). Clipping was performed at the beginning of each grazing period to determine forage availability and to ensure that the results were due to the treatments offered (CON and BAC) and not due to potential differences in forage availability between the paddocks. Clipped samples were dried in the microwave for DM determination. Herbage mass was calculated by recording the DM weight per 1 m^2^ from the clipped sample and converted to kg of DM/ha for each paddock. Similarly to the aforementioned, clipped samples at the beginning and end of each grazing period were analyzed for the nutritional profile. Moreover, DM disappearance from each paddock was estimated by subtracting the initial–final DM remaining over each 15-d grazing period.

Individual full BW was collected on days -1 (end of the dry season), 0, 1, 3, 7, 14, and 15 of each grazing period, whereas initial and final BW were determined as the mean BW on days -1 and 0 and 14 and 15, respectively. Blood samples were collected on days 0, 1, 3, 7, and 14 of the grazing period for determination of serum haptoglobin (Cooke and Arthington 2013), IGF-I (#SG100; R&D Systems, Inc., Minneapolis, MN; Cooke et al. 2012), BUN and total serum protein (**TSP**; BS200E Veterinary Chemistry Analyzer; Mindray, California, USA), Na and K (XI-921 electrolyte analyzer; Pioway medical lab equipment Co., Nanjing, China), glucose, non-esterified fatty acids (**NEFA**), and beta-hydroxybutyrate (**BHBA**; respons®910VET Veterinary Chemistry Analyzer; DiaSys Diagnostic Systems USA LLC, Wixom, MI; Colombo et al. 2021). The intra- and inter-assay CV for these analyses were ≤ 8.2%.

Lastly, fecal samples were manually collected from the rectum of each heifer to assess fecal pH immediately following collection using a hand-held pH meter (AET15 pH tester, Fisher Scientific; Dias e Silva et al. 2023). Fecal scores (1 = solid; 2 = soft; 3 = watery diarrhea; Renaud et al. 2020) were recorded once daily at 0800 h for each heifer from days 0 to 15, while heifers were either on pasture or in the working facility for additional blood and BW sampling. Fecal scores were also utilized to calculate the cumulative incidence of diarrhea (heifers with fecal score 3), the percentage of heifers with no diarrhea, 1 d of diarrhea, and ≥ 2 d of diarrhea (prolonged diarrhea), and the total days of diarrhea symptoms (days of fecal score 3), as described by Souza et al. (2025).

### Statistical analysis

For both experimental phases, all data were analyzed using the individual heifer as the experimental unit with the SAS Statistical Software (version 9.4; SAS Inst. Inc., Cary, NC). For all statistical analyses, significance was set at *P* ≤ 0.05, whereas tendencies were denoted if 0.05 < *P* ≤ 0.10.

*Dry season.* Dry matter intake data collected prior to the beginning of the experimental period (days -14 to -1), BW, blood samples, and ATTD data obtained prior to treatment administration were used as a covariate for all performed herein. All data (initial and final BW, BW change, ADG, nutrient digestibility, PSPS, and blood hormones and metabolites) were analyzed with PROC MIXED of SAS (SAS Inst.), considering treatment, day, and the resulting interaction as fixed effects, while heifer was used as the random factor. All results were covariately-adjusted, with the exception being the PSPS values, and reported according to the main order effect.

*Early vegetative season.* The nutritional profile of the paddock at the beginning and end of the 15-d grazing period, as well as DM disappearance of each paddock was analyzed using treatment as fixed effect, whereas paddock(treatment) was included as the random effect in the model. Individual BW of the heifers, pH, and blood variables were analyzed as repeated measurements of the SAS (PROC MIXED; SAS Inst.), considering heifer as the experimental unit, treatment, day, and the resulting interaction as fixed effects. Day was used in the repeated statement, whereas heifer(day) was the subject, and autoregressive-1 was the covariate structure for all repeated measures analyses based on the AIC. The cumulative incidence of diarrhea and percentage of heifers with 0, 1, and ≥ 2 d of diarrhea were analyzed using the GLIMMIX procedure of SAS.

## Results

### Dry season

No treatment × day interactions (*P* ≥ 0.11) were observed for any of the variables analyzed herein; hence, only main treatment effects will be presented. No treatment effects were observed for forage, supplement, or forage + supplement intake (*P* ≥ 0.23; Table 2). On the other hand, apparent total-tract of DM and CP digestibility was greater for heifers fed BAC (*P* ≤ 0.05; Table 2), but no effects were observed for apparent total-tract NDF, ADF, and hemicellulose digestibility (*P* ≥ 0.38). Table 3 demonstrates the proportion of the hay retained in the different sieves of the PSPS assessed during the experimental period. Heifers fed BAC were offered and, thus, consumed low-quality tropical hay with a lower proportion of particles larger than 8 (*P* = 0.05) and 19 mm (*P* = 0.03), as well as higher proportion of 4 mm particles and bottom sieves (*P* = 0.04; Table 3) when compared with CON cohorts.

**Table 2:** Forage, supplement, and total intake, as well as apparent total-tract digestibility (**ATTD**) of nellore beef heifers receiving a low-quality tifton 85 (*Cynodon* sp.) hay and a protein-based supplement containing or not (**CON**; *n* = 24) a *bacillus*-based DFM (**BAC**; *n* = 24)^a^.

Item	Treatments		SEM	*P*-value
	CON	BAC		
**Intake, kg/d^b^**				
** Forage**	6.76	6.60	0.100	0.23
** Supplement**	0.90	0.92	0.029	0.65
** Total**	7.66	7.52	0.199	0.73
**Apparent total-tract digestibility, %^c^**				
** Dry matter**	72.8	75.8	1.35	0.05
** Crude protein**	35.6	41.2	1.90	0.04
** Neutral detergent fiber**	53.4	53.2	0.77	0.87
** Acid detergent fiber**	32.4	31.7	0.54	0.38
** Hemicellulose**	20.2	20.3	0.34	0.75

a Heifers were fed daily a Tifton 85 hay to ensure ad libitum intake, whereas the protein-based supplement (Tecnophós Proteico EN-25; TecnoBeef Indústria e Comércio S.A., Altair, SP, Brazil) was offered at 0.3% of heifer body weight. Heifers were assigned to receive the protein-based supplement with or without (**CON**) the addition of a *Bacillus*-based DFM containing *B. licheniformis* 809 and *B. subtilis* 810 (**BAC**; Bovacillus, Novonesis, Valinhos, SP, Brazil).

b Intake was measured daily for 140 d.

c Fecal samples were collected for the last 3 d of each 28-d experimental period.

**Table 3:** Particle distribution (% of hay retained) of the low-quality tifton 85 (*cynodon* sp.) hay offered to nellore heifers receiving a protein-based supplement containing or not (**CON**; *n* = 24) a *bacillus*-based DFM (**BAC**; *n* = 24)^a^^,^^b^.

Item	Treatments		SEM	*P*-value
	CON	BAC		
**% of hay retained**				
** 19-mm**	28.9	24.4	1.58	0.05
** 8-mm**	26.5	23.5	0.93	0.05
** 4-mm**	15.7	18.1	0.86	0.04
** Bottom pan**	28.9	34.0	1.70	0.04

a Heifers were fed daily a Tifton 85 hay to ensure ad libitum intake, whereas the protein-based supplement (Tecnophós Proteico EN-25; TecnoBeef Indústria e Comércio S.A., Altair, SP, Brazil) was offered at 0.3% of heifer body weight. Heifers were assigned to receive the protein-based supplement with or without (**CON**) the addition of a *Bacillus*-based DFM containing *B. licheniformis* 809 and *B. subtilis* 810 (**BAC**; Bovacillus, Novonesis, Valinhos, SP, Brazil).

b Penn State Particle Separator was determined according to the methodology described by Kononoff and Heinrichs (2003).

Table 4 reports the performance results of the heifers during the experimental period. No treatment effects were observed on initial and final BW, BW change, and ADG during the dry season (*P* ≥ 0.21). Treatment effects were observed on blood urea-N and plasma IGF-I (*P* ≤ 0.02), while trends were observed for plasma glucose (*P* = 0.09). Beef heifers fed BAC had a greater mean plasma BUN and IGF-I vs. CON cohorts. Moreover, mean plasma glucose tended to be greater for heifers fed BAC vs. CON, while no effects were observed for plasma haptoglobin (*P* ≥ 0.81; Table 5).

**Table 4:** Performance results of Nellore beef heifers receiving a protein-based supplement containing or not (**CON**; *n* = 24) a *Bacillus*-based DFM (**BAC**; *n* = 24) during the dry season^a^^,^^b^.

Item	Treatments		SEM	*P*-value
	CON	BAC		
**Body weight, kg**				
** Initial**	316	310	4.8	0.38
** End of the dry season**	342	344	5.9	0.83
**Body weight change, kg**	26	34	4.4	0.21
**Average daily gain, kg**	0.185	0.241	0.0309	0.21

a Heifers were fed daily a Tifton 85 hay to ensure ad libitum intake, whereas the protein-based supplement (Tecnophós Proteico EN-25; TecnoBeef Indústria e Comércio S.A., Altair, SP, Brazil) was offered at 0.3% of heifer body weight. Heifers were assigned to receive the protein-based supplement with or without (**CON**) the addition of a *Bacillus*-based DFM containing *B. licheniformis* 809 and *B. subtilis* 810 (**BAC**; Bovacillus, Novonesis, Valinhos, SP, Brazil).

b Dry season lasted 140 d.

**Table 5:** Metabolic and physiological responses of Nellore beef heifers receiving a protein-based supplement containing or not (**CON**; *n* = 24) a *bacillus*-based DFM (**BAC**; *n* = 24) during the dry season^a^^,^^b^^,^^c^.

Item	Treatments		SEM	*P*-value
	CON	BAC		
**Blood urea-N, mg/dL**	12.1	11.3	0.23	0.02
**Glucose, mg/dL**	63.6	69.1	2.28	0.09
**Insulin like-growth factor-I, ng/mL**	121	137	3.6	< 0.01
**Haptoglobin, mg/mL**	0.179	0.171	0.0234	0.81

a Heifers were fed daily a Tifton 85 hay to ensure ad libitum intake, whereas the protein-based supplement (Tecnophós Proteico EN-25; TecnoBeef Indústria e Comércio S.A., Altair, SP, Brazil) was offered at 0.3% of heifer body weight. Heifers were assigned to receive the protein-based supplement with or without (**CON**) the addition of a *Bacillus*-based DFM containing *B. licheniformis* 809 and *B. subtilis* 810 (**BAC**; Bovacillus, Novonesis, Valinhos, SP, Brazil).

b Dry season lasted 140 d.

c Blood samples were taken every 28 d.

### Grazing (early vegetative) season

**Figure 1:**
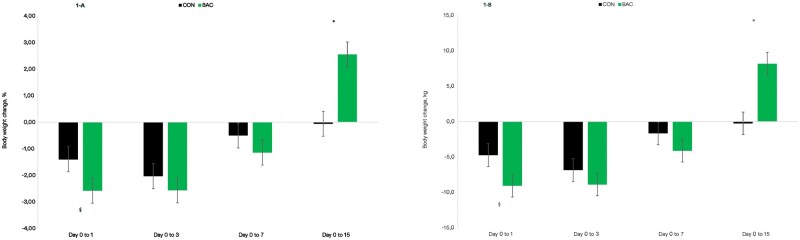
Body weight change, as a % (A) or kg (B), of Nellore beef heifers receiving a protein-based supplement containing or not (**CON**; *n* = 24) a *bacillus*-based DFM (**BAC**; *n* = 24) during the 15-d grazing period. A treatment × day interaction was observed for both variables (*P* < 0.001). *Denotes significance at *P* ≤ 0.05 level, whereas § denotes significance at 0.05 > *P* ≤ 0.10.

No differences were observed on fecal pH (*P* = 0.44; data not shown) and no diarrhea occurrence was observed in the current study (data not shown) and, therefore, diarrhea incidence during the study was not analyzed. Table 6 reports the average performance results of heifers during the 15-d grazing season and for the entire experimental period (dry + grazing seasons). Heifers fed BAC had a greater (*P* ≤ 0.02) BW change and ADG during both periods of assessment, grazing season and total experimental period. Body weight change was 8.4 and 16.2 kg greater for BAC during the grazing season and for the entire experimental period, respectively, whereas ADG was 562 and 104 g greater during the same periods, respectively. Treatment × day interactions were observed on BW change, reported either as % or kg, during the 15-d grazing season (*P* < 0.01; Fig. 1A and B). For both analyses, heifers fed BAC tended to lose more BW (*P* ≤ 0.09) from day 0 to 1, but gained more BW from day 0 to 15 (*P* > 0.01) of the grazing period. No further differences were observed in the other sampling days (*P* ≥ 0.34).

**Table 6:** Performance results of nellore beef heifers receiving a protein-based supplement containing or not (**CON**; *n* = 24) a *bacillus*-based DFM (**BAC**; *n* = 24) during the grazing season and for the entire experimental period^a^^,^^b^.

Item	Treatments		SEM	*P*-value
	CON	BAC		
**Body weight, kg**				
** End of the early growth season**	342	352	5.4	0.05
**Body weight change, kg**				
** Dry to early growth season**	−0.254	8.154	2.124	<0.01
** Total**	25.9	42.1	4.6	0.02
**Average daily gain, kg**				
** Dry to early growth season**	−0.018	0.544	0.141	<0.01
** Total^c^**	0.166	0.270	0.030	0.02

a Heifers were fed daily a Tifton 85 hay to ensure ad libitum intake, whereas the protein-based supplement (Tecnophós Proteico EN-25; TecnoBeef Indústria e Comércio S.A., Altair, SP, Brazil) was offered at 0.3% of heifer body weight. Heifers were assigned to receive the protein-based supplement with or without (**CON**) the addition of a *Bacillus*-based DFM containing *B. licheniformis* 809 and *B. subtilis* 810 (**BAC**; Bovacillus, Novonesis, Valinhos, SP, Brazil).

b Grazing season lasted 15 d.

c Includes the beginning of the dry to the end of the grazing season (156 d).

Table 7 reports the DM availability and nutritional profile of the Tifton 85 paddocks used during the 15-d grazing period. No treatment effects were observed on availability of DM of the paddocks at the beginning of the grazing period (*P* = 0.61), whereas paddocks assigned to heifers fed BAC had less DM available at the end of the 15-d grazing period (*P* = 0.02; Table 7) and a greater estimated DM disappearance from the paddocks (*P* = 0.04). No differences were observed on the nutritional profile of the paddocks at either beginning (*P* ≥ 0.14) or end (*P* ≥ 0.28) of the grazing period.

**Table 7:** Dry matter availability, disappearance, and nutritional profile of the tifton 85 (*Cynodon* sp.) paddocks assigned to Nellore beef heifers receiving a protein-based supplement containing or not (**CON**; *n* = 24) a *bacillus*-based DFM (**BAC**; *n* = 24) during the grazing season^a^^,^^b^.

Item	Treatments		SEM	*P*-value
	CON	BAC		
**Dry matter availability,^c^ kg**				
** Day 0**	150.7	157.5	12.30	0.61
** Day 15**	144.1	105.9	14.05	0.02
**Dry matter disappearance, kg/d**	0.44	3.44	1.295	0.04
**Grass nutritional profile**				
** Day 0**				
** Dry matter, %**	19.3	16.6	1.81	0.28
** Ash, % DM**	11.8	12.0	0.23	0.58
** Crude protein, % DM**	16.5	16.9	0.47	0.51
** Non-protein N, % CP**	0.98	0.96	0.035	0.63
** Ether extract, % DM**	1.44	1.30	0.068	0.14
** Neutral detergent fiber, % DM**	54.7	55.0	1.11	0.88
** Acid detergent fiber, % DM**	33.3	34.4	0.59	0.23
** Hemicellulose, % DM**	21.4	20.6	0.77	0.48
** Non-fibrous carbohydrate, % DM**	15.6	14.8	0.64	0.39
** Total digestible nutrients, % DM**	58.5	58.5	0.15	0.95
** Day 15**				
** Dry matter, %**	13.9	14.0	0.71	0.94
** Ash, % DM**	9.60	9.90	0.234	0.37
** Crude protein, % DM**	12.0	12.0	0.39	0.95
** Non-protein N, % CP**	0.83	0.88	0.036	0.34
** Ether extract, % DM**	1.45	1.41	0.050	0.56
** Neutral detergent fiber, % DM**	61.5	60.8	1.12	0.67
** Acid detergent fiber, % DM**	35.5	36.1	0.63	0.57
** Hemicellulose, % DM**	25.9	24.6	0.88	0.30
** Non-fibrous carbohydrate, % DM**	15.8	16.0	0.77	0.82
** Total digestible nutrients, % DM**	58.9	58.6	0.20	0.28

a Heifers were fed daily a Tifton 85 hay to ensure ad libitum intake, whereas the protein-based supplement (Tecnophós Proteico EN-25; TecnoBeef Indústria e Comércio S.A., Altair, SP, Brazil) was offered at 0.3% of heifer body weight. Heifers were assigned to receive the protein-based supplement with or without (**CON**) the addition of a *Bacillus*-based DFM containing *B. licheniformis* 809 and *B. subtilis* 810 (**BAC**; Bovacillus, Novonesis, Valinhos, SP, Brazil).

b Grazing season lasted 15 d.

c Dry matter availability was estimated immediately before placing the heifers in the paddocks (day 0) and at the end of the 15-d grazing period.

Lastly, Table 8 reports the metabolic and physiological responses of the beef heifers during the grazing season. Heifers fed BAC had a lower (*P* = 0.05) mean plasma concentration of potassium and greater (*P* = 0.05) mean plasma glucose compared with CON heifers. No further effects were observed in the other plasma variables (*P* ≥ 0.24), whereas day effects were observed for all blood variables analyzed herein (*P* < 0.01; Supplementary Fig. 1).

**Table 8:** Metabolic and physiological responses of nellore beef heifers receiving a protein-based supplement containing or not (**CON**; *n* = 24) a *bacillus*-based DFM (**BAC**; *n* = 24) during the grazing season^a^^,^^b^.

Item	Treatments		SEM	***P*-value** ^c^		
	CON	BAC		*T*	*D*	*T × D*
**Blood urea-N, mg/dL**	18.3	18.9	0.49	0.55	< 0.001	0.75
**Glucose, mg/dL**	43.8	51.1	3.25	0.05	< 0.01	0.47
**Beta-hydroxybutyrate, mg/dL**	3.18	3.18	0.110	0.97	< 0.01	0.83
**Potassium, mmol/L**	6.14	5.81	0.116	0.05	< 0.001	0.68
**Sodium, mmol/L**	138.5	135.2	1.91	0.24	< 0.001	0.93
**Sodium to potassium ratio**	22.6	23.2	0.44	0.63	< 0.001	0.80
**Non-esterified fatty acids, mEq/L**	0.270	0.273	0.0128	0.88	< 0.001	0.75
**Osmolality, mOsm/kg**	287	282	3.9	0.34	< 0.001	0.86
**Total protein, g/dL**	6.39	6.17	0.157	0.32	< 0.001	0.98
**Insulin-like growth fator-1, ng/mL**	192	188	7.4	0.71	< 0.0001	0.95
**Haptoglobin, mg/mL**	0.516	0.469	0.091	0.72	< 0.0001	0.84

a Heifers were fed daily a Tifton 85 hay to ensure ad libitum intake, whereas the protein-based supplement (Tecnophós Proteico EN-25; TecnoBeef Indústria e Comércio S.A., Altair, SP, Brazil) was offered at 0.3% of heifer body weight. Heifers were assigned to receive the protein-based supplement with or without (**CON**) the addition of a *Bacillus*-based DFM containing *B. licheniformis* 809 and *B. subtilis* 810 (**BAC**; Bovacillus, Novonesis, Valinhos, SP, Brazil).

b Blood samples were taken on days 0, 1, 3, 7, and 14 of the experimental period.

c **T** = treatment effect; **D** = day effect; **T × D** = treatment × day interaction.

## Discussion

The main goals of the current experiment were to evaluate the effects of a *Bacillus*-based DFM, containing a combination of *B. licheniformis* 809 and *B. subtilis* 810, supplementation on (i) feed intake, apparent total-tract nutrient digestibility, growth rates, and metabolic responses of *B. indicus* beef heifers offered a low-quality warm season hay (<5% CP) and a protein-based supplement during the dry season and (ii) on growth rates and metabolic responses of the same beef heifers grazing tropical forages during the early vegetative stage for 15 d.

### Dry season

*Bacillus* spp. has been reported to produce a wide array of digestive enzymes including cellulases, expansin-like proteins, proteases, and xylanases (Schallmey et al. 2004; Liu et al. 2015; Pech-Cervantes et al. 2019; Luise et al. 2022), which, in turn, could benefit ruminants grazing forages with considerable amounts of structural carbohydrates. In vitro DM and NDF digestibility improved when a DFM containing *B. licheniformis* 809 and *B. subtilis* 810 were incubated with forages of varying qualities (Pan et al. 2022; Cappellozza et al. 2023), as well as when dairy TMR diets were assessed (Cappellozza et al. 2023). Hence, it is plausible to speculate that their supplementation to beef cattle grazing low-quality tropical forages would lead to a greater nutrient digestibility and growth rates. This statement is supported by the fact that, in cattle grazing low-quality grasses, the limitation of feed intake and growth is likely caused by the greater NDF content of the forages, slow passage rate, and the resulting ruminal filling effect (Mertens 1994; Allen and Piantoni 2013; NASEM 2016). Contrary to our hypothesis, we have not observed treatment effects on forage and forage + concentrate intake, apparent total-tract NDF or ADF digestibility, but heifers fed *Bacillus* spp. had greater apparent DM and CP total-tract digestibility. Our results partially agree with Limede et al. (2025), who reported greater apparent total-tract DM, CP, NDF, and ADF digestibility, resulting in greater forage intake in rumen-fistulated beef cows offered a medium-quality hay (8.8% CP) and *B. licheniformis* 809 and *B. subtilis* 810 mixed with DDGS for approximately 30 d. Beef steers fed a 70% NDF Rhodes-grass hay and ground barley mixed with *B. licheniformis* 809 and *B. subtilis* 810 also had a greater total-tract DM and NDF digestibility, but no differences were observed on forage intake (Eyre et al. 2025). Moreover, Souza et al. (2026) also observed greater DM and CP digestibility of beef heifers fed a low-quality tropical hay and concentrate with the addition of *B. licheniformis* 809 and *B. subtilis* 810, without effects on feed intake. Hence, the fact that DMI was not increased in the current study may be explained by the lack of effects on NDF and ADF digestibility, even though DM and CP digestibility were positively impacted by BAC.

One of the possible explanations for the lack of treatment effects on apparent total-tract NDF and ADF digestibility and forage intake might be related to the particle size of the low-quality tropical roughage assessed by the PSPS during the experiment. As reported herein, BAC heifers received tropical forages with smaller particle sizes than unsupplemented heifers, which could have limited the potential effects on nutrient digestibility, as smaller particles likely reach the liquid phase of the rumen, whereas most of the fiber degradation occurs in the mat (or solid) phase of the rumen (NASEM 2016). This point likely limited (i) the rumen filling effect often expected when low-quality grasses are offered to the herd and (ii) the potential impact of the BAC in the NDF and ADF digestibility. On the other hand, the fact that *Bacillus* spp. also secretes proteases (Luise et al. 2022) may explain the greater CP digestibility observed herein and by others (Limede et al. 2025; Souza et al. 2026) when greater amounts of concentrates were offered to the herd (≥500 g/head per day). Recently, Detmann et al. (2024) reviewed the impacts of nutrient digestibility and, more specifically, NDF digestibility on performance of beef cattle grazing tropical forages. These authors demonstrated that total-tract NDF digestibility is highly correlated with the rumen degradation of this nutrient, suggesting that nutritional alternatives that maximize NDF degradation in the rumen will also lead to a greater total-tract NDF digestibility, voluntary forage intake, and performance of the beef cattle herd (Detmann et al. 2024). Moreover, greater rumen NDF digestibility resulted in a greater rumen microbial N production, likely explained by the greater amount and availability of energy for microbial synthesis (Detmann et al. 2024). Tropical forages often present greater amount of fiber, as well as reduced concentrations of protein (total and available) and non-structural carbohydrates compared with temperate grasses of similar nutritional value (Wilson et al. 1983; Barbehenn and Bernays 1992). Indeed, Bohnert et al. (2011) demonstrated that tropical grasses also have a lower rate of ruminal DM degradation, lower effective degradability of DM, NDF, and N, as well as a greater undigestible nutrient fraction when compared with temperate grasses. Hence, the utilization of nutritional strategies that optimize forage intake and nutrient degradation is paramount to optimize performance of cattle grazing tropical grasses, as demonstrated by others under metabolic and controlled settings (Bohnert et al. 2011).

Previous studies demonstrated that supplementation of BAC has improved growth rates of pre-weaning calves (Magalhães et al. 2024; Copani et al. 2025; Terré et al. 2025) and newly-weaned grazing beef steers (Mackey et al. 2024), feedlot cattle (Dias et al. 2022; Cordeiro et al. 2024), as well as positively altered the pre-calving BCS of grazing pregnant heifers (Izquierdo et al. 2024) and their progenies (Izquierdo et al. 2024; Moriel et al. 2025). The effects of BAC leading to greater growth rates likely involve, but are not limited to, modulation of the immune response (Mackey et al. 2024, 2025; Bailey et al. 2025; Terré et al. 2025), enzymatic activity and greater nutrient degradation (Pan et al. 2022; Cappellozza et al. 2023; Limede et al. 2025; Souza et al. 2026), modulation of the metabolic pathways associated with energy, protein, and immunity (Oyebade et al. 2024; Izquierdo et al. 2025), fecal microbiome (Sousa et al. 2025), rumen microbiota (Lamontagne et al. 2023; Linde et al. 2023), and health-supportive effects in the herd (Mackey et al. 2024, 2025; Magalhães et al. 2024; Capern et al. 2025; Terré et al. 2025). Nonetheless, we have not reported a significant effect on BW change and ADG of *B. indicus* beef heifers offered a low-quality tropical grass and a protein-based supplement, although a numerical improvement was seen for both BW change (+ 8.0 kg) and ADG (+ 56 g), which can be likely explained by the greater SEM. Mackey et al. (2024) reported lower morbidity and mortality rates in high-risk stocker cattle fed BAC, which positively impacted the BW change of the animals available throughout the study. On the other hand, Bailey et al. (2025) recently reported no significant effects of BAC supplementation on ADG and final BW of newly-weaned beef heifers grazing tall fescue forages, although the concentration of pro-inflammatory cytokines was reduced in the group of heifers fed BAC, indicating a modulation of the immune response by BAC. However, few differences between studies should be considered and discussed when interpreting the current results vs. previous data available in the literature: (i) heifers were housed individually during the current study, which likely impacts the ingestive behavior (Neave et al. 2018) and growth rates of the herd, (ii) the fact that the study was designed to evaluate the effects of BAC on nutrient digestibility of the beef heifers offered a low-quality forage over an extended period of time during the dry season (≥140 d), and (iii) no noted stressor was adopted and/or used as a challenge model herein.

Even though no significant effects were reported on ADG and BW change of beef heifers during the dry season, BAC feeding reduced plasma BUN, while increasing mean plasma glucose and IGF-I. The exact mechanisms leading to these responses is currently unknown, but might be associated with a greater synchrony of energy and N in the rumen, given that rumen VFA and nutrient degradation have been positively impacted by *Bacillus* spp. supplementation (Dias et al. 2022; Souza et al. 2026) and/or a greater synthesis of microbial protein in the rumen and better intestinal absorption (NASEM 2016), also agreeing with Detmann et al. (2024). Moreover, the fact that plasma glucose and IGF-I were positively impacted by BAC feeding suggests that energy metabolism was also altered, agreeing with previous research in beef (Izquierdo et al. 2024; Souza et al. 2026) and dairy cattle (Cappellozza et al. 2024; Arthur et al. 2025). Previous studies also demonstrated that supplementing a combination of *B. licheniformis* 809 and *B. subtilis* 810 reduced rumen ammonia-N in beef animals offered a high-concentrate diet (Dias et al. 2022; Cordeiro et al. 2024), reduced BUN in beef animals offered low-quality temperate and tropical grasses (Limede et al. 2025; Souza et al. 2026), blood metabolomic markers associated with protein metabolism in pregnant heifers and their progenies (Izquierdo et al. 2025), reduced BUN and milk urea-N in late-lactating dairy cows (Arthur et al. 2025), and increased efficiency of protein utilization in dairy cows (Terré et al. 2024; Bach et al. 2026).

### Grazing (early vegetative) season

In Europe, beef calves with summer scour syndrome following their first exposure to high-protein grasses also had high blood ammonia levels (Here et al. 2024), suggesting a role of N and/or its metabolites in the occurrence of such syndrome. In the tropics, Souza et al. (2025) developed a feed restriction model to test the hypothesis that cattle grazing early vegetative forages would present diarrhea, performance losses, and metabolic alterations that could explain the underlying mechanisms initially described by Here et al. (2024). In fact, greater BUN levels were reported in beef heifers that had diarrhea cases with greater severity during some days of the experiment (Souza et al. 2025). Contrary to what was hypothesized, we have not observed any cases of diarrhea during the 15-d grazing period adopted herein, even though the CP concentration of the forages was comparable to Here et al. (2024), but lower than the CP concentration in Souza et al. (2025; approximately 25% on a DM basis). Nonetheless, Souza et al. (2025) also adopted a feed restriction model that, in addition to the CP concentration of the forages, likely caused a neuroendocrine stress and resulted in the release of pro-inflammatory compounds, such as pro-inflammatory cytokines and reactive oxygen species, that might have compromised the integrity of intestinal epithelial cells (Boll et al. 2024a, 2024b). However, the rationale of not adopting the same model developed by Souza et al. (2025) was to mimic a commercial setting, where the grazing herd usually has access to a low-quality forage during the dry season in the tropics and, following the first rain events, transitions to paddocks with lush forages in the early vegetative stage.

Although no diarrhea occurrence was reported herein, all heifers, regardless of treatment, experienced significant BW losses in the first days of the grazing period, indicating that the nutritional profile of the forage likely altered the rumen fermentation, passage rate of liquids and solids, as well as the metabolism of the animals, resulting in BW losses. Nevertheless, by the end of the 15-d grazing period, heifers fed BAC gained more BW when compared with CON cohorts, suggesting that the recovery of the initial BW loss was faster and greater in heifers fed the *Bacillus*-based DFM. Moreover, the disappearance of DM was greater in paddocks with BAC vs. CON heifers and the changes in nutrient profile between the paddocks may indicate a greater forage DM and nutrient intake during the grazing period, supporting the rationale that under challenging situations, BAC supplementation positively impacts the performance of the beef and dairy cattle herd (Mackey et al. 2024; Magalhães et al. 2024; Terré et al. 2025). Nonetheless, additional studies are warranted to accurately measure the DMI and long-term performance of beef cattle exposed to lush pastures, similar to what has been reported by Souza et al. (2025).

The excessive intake of protein and, more specifically, non-protein N (i.e., urea) in the supplement and lush forages likely induces diarrhea in ruminants (Here et al. 2024; Souza et al. 2025). The possible causes for this digestive upset include, but may not be limited to, an excessive accumulation of ammonia-N in the rumen that may lead to an increase in the rumen pH (Nolan 2003; Huntington and Archibeque 2000), increased osmolality in the rumen fluid causing excessive fluid accumulation in the GIT (Owens and Goetsch 1986), impaired rumen function (Russell and Diez-Gonzalez 1998) that allows a greater amount of undigested dietary protein to be further digested in the intestines (Huntington and Archibeque 2000), and electrolytes imbalances (Owens and Goetsch 1986; Constable 2003). In fact, BAC heifers had reduced plasma concentrations of potassium when compared with CON cohorts, which could be an indication of diarrhea occurrence, but no diarrhea cases were reported herein. Moreover, heifers fed BAC had a greater plasma glucose concentration, likely explained by the greater growth rates (DM disappearance, ADG, and BW change) following the 15-d grazing period, as plasma glucose is associated with the nutrient intake and performance of beef cattle (Vizcarra et al. 1998; Cappellozza et al. 2014a, 2014b). These results agree with previous authors demonstrating that *B. licheniformis* 809 and *B. subtilis* 810 supplementation increased plasma glucose in crossbred calves presenting a greater feed intake and feed efficiency (Terré et al. 2025), late-lactating dairy cows with an improved feed efficiency (Arthur et al. 2025), rumen-fistulated beef heifers (Souza et al. 2026), and pregnant grazing beef heifers (Izquierdo et al. 2024). Izquierdo et al. (2024) also demonstrated that, besides the improvement in plasma glucose, heifers fed BAC during pre-partum also had a greater BCS change (+ 0.31 BCS) when compared with cohorts not fed BAC. Moreover, in ruminants, plasma glucose is primarily produced from propionate production in the rumen (Bergman 1990) and BAC has also been reported to modulate rumen fermentation, favoring propionate formation (Maderal et al. 2022; Linde et al. 2023; Souza et al. 2026). Hence, it can be speculated that following the exposure to lush pastures and excessive BW loss, cattle fed BAC were able to resume feed intake and, likely, nutrient digestion and microbial community (Lamontagne et al. 2023; Linde et al. 2023) faster than CON heifers, that optimized the ADG of the herd over the 15-d grazing period. Recently, Mackey et al. (2025) reported a faster resumption of feed intake in beef heifers administered lipopolysaccharide and fed *B. licheniformis* 809 and *B. subtilis* 810. Altogether, these positive effects and distinct modes of action reported herein and extensively by others (Luise et al. 2022; Cappellozza et al. 2025) likely contributed to the greater overall ADG and BW change during the entire experimental period, comprising both the dry and grazing periods.

In summary, feeding *Bacillus licheniformis* 809 and *B. subtilis* 810 benefited dry matter and crude protein digestibility, and plasma hormones and metabolites associated with energy and protein metabolism in beef heifers fed a low-quality tropical hay and a protein-based concentrate, without effects on ADG and BW change of the herd. Nonetheless, when the heifers were transitioned to early vegetative pastures for 15 d, *Bacillus* spp. supplementation improved the ADG and BW change of those heifers, while also impacting plasma potassium and glucose. Additional studies are warranted to understand the effects of *B. licheniformis* 809 and *B. subtilis* 810 supplementation on long-term period performance and health of *Bos indicus* cattle grazing lush pastures for longer period of time.

## Supplementary Material

txag046_Supplementary_Data

## Abbreviations

ADF: Acid detergent fiber
ADG: Average daily gain
ATTD: Apparent total-tract digestibility
BAC: *Bacillus*-based DFM treatment group
BHBA: ß-hydroxybutyrate
BUN: Blood urea-N
BW: Body weight
CON: Control treatment group
CP: Crude protein
DFM: Direct-fed microbial
DM: Dry matter
DMI: Dry matter intake
FE: Feed efficiency
GIT: Gastrointestinal tract
IGF-I: Insulin-like growth factor 1
NDF: Neutral detergent fiber
NEFA: Non-esterified fatty acids
PSPS: Penn State Particle Separator
TDN: Total digestible nutrients
TP: Total protein
