# Supplementary material for: Effects of supplementing a Bacillus-based direct-fed microbial on feed intake, apparent total-tract nutrient digestibility, growth rates, and metabolic responses of Bos indicus beef heifers during the dry and early vegetative seasons
Source: Transl Anim Sci. 2026 Apr 22;10:txag046. doi: 10.1093/tas/txag046 (PMC13175177; doi:10.1093/tas/txag046)
Supplement: txag046_Supplementary_Data [file txag046_supplementary_data.docx]

**Supplementary Figure 1.** Plasma concentrations of hormones and metabolites in *Bos indicus* beef heifers during the grazing period. Day effects were detected (*P* ≤ 0.01) for blood urea-N (**BUN**; mg/dL), glucose (mg/dL), β-hydroxybutyrate, (**BHBA**; mg/dL), potassium (mmol/L), sodium (mmol/L), sodium to potassium ratio, non-esterified fatty acids (**NEFA**; mEq/L), haptoglobin (mg/mL), potassium (mmol/L), total protein (**TP**; g/dL), osmolality (mOsm/kg), and insulin-like growth-factor-I (**IGF-I**; ng/mL).
